# Supplementary material for: Exercise training improves exercise capacity independent of AMPKα2 T172-mediated adaptations in skeletal muscle
Source: bioRxiv. 2026 Jun 23:2026.06.18.733224. Preprint. [Version 1] doi: 10.64898/2026.06.18.733224 (PMC13320912; doi:10.64898/2026.06.18.733224)
Supplement: Supplement 1 [file NIHPP2026.06.18.733224v1-supplement-1.pdf]

bioRxiv preprint doi: https://doi.org/10.64898/2026.06.18.733224; this version posted June 23, 2026. The copyright holder for this preprint (which was not certified by peer review) is the author/funder, who has granted bioRxiv a license to display the preprint in perpetuity. It is made available under a CC-BY 4.0 International license.

## Supplementary information/figures

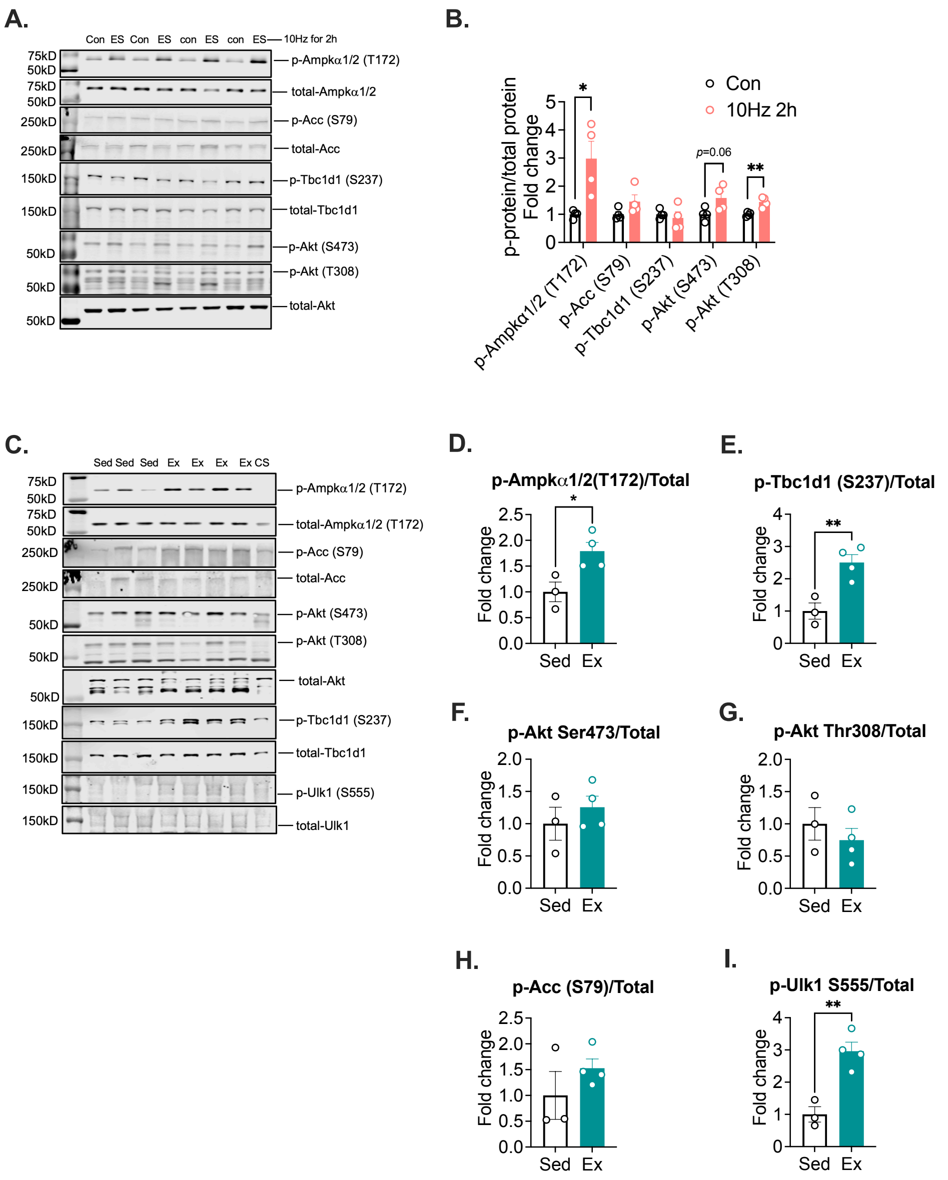


**Supplementary figure 1. Acute treadmill running and electrical stimulation activate Ampkα T172 in skeletal muscle.** Wild type C57BL/6J mice (male) were subjected to 90 min acute treadmill running with sedentary mice as control or unilateral electrical stimulation of the sciatic nerve at 10 Hz for 2 hours with the contralateral leg as control. Plantaris muscles were analyzed by western blot. **A**: Representative western blot images for p-Ampkα1/2 (T172), p-Acc (S79), p-Tbc1d1 (S237), p-Akt (S473 & T308), and respective total proteins in electrically stimulated muscle (ES) and the contralateral control muscle (Con); **B**: Quantification of protein expression, n = 4; **C**: Representative western blot images for p-Ampkα1/2 (T172), p-Acc (S79), p-Akt (S473 & T308), p-Tbc1d1 (S237), p-Ulk1 (S555), and respective total proteins from exercised (Ex) and sedentary mice (Sed), CS stands for common standard; **D-I**: Quantification of protein expression, n = 3-4 per group. Data presented as means ± SEM and analyzed by two-tailed *t*-test. *$p < 0.05$, **$p < 0.01$.

bioRxiv preprint doi: https://doi.org/10.64898/2026.06.18.733224; this version posted June 23, 2026. The copyright holder for this preprint (which was not certified by peer review) is the author/funder, who has granted bioRxiv a license to display the preprint in perpetuity. It is made available under a CC-BY 4.0 International license.

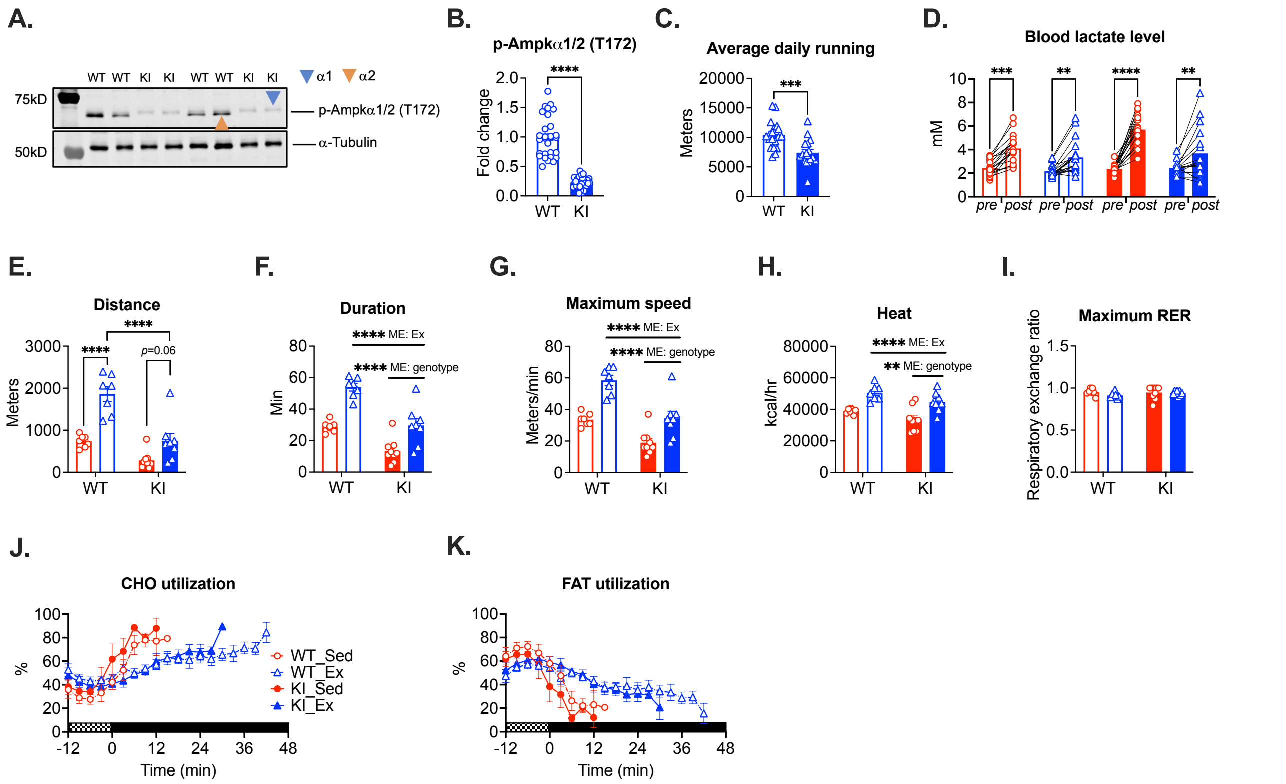


**Supplementary figure 2. Additional parameters measured during exhaustive treadmill running test and $VO_2$max treadmill running test.** *Ampkα2*(T172A) KI and WT littermates were subjected to VWR for 4 weeks with sedentary KI and WT mice as controls followed by exhaustive treadmill running test and $VO_2$max treadmill running test. **A**: Representative western blot image for p-Ampkα1/2 (T172) with α-tubulin as loading control in plantaris muscles. Blue triangle points to α1 subunit, and orange triangle points to α2 subunit; **B**: Quantification of the p-Ampkα1/2 (T172) in WT and *Ampkα2*(T172) KI mice; **C**: Average daily voluntary wheel running distance for WT and KI mice, n = 18-20 per group. Data presented as means ± SEM and analzyed by two-tailed *t*-test. *** $p < 0.001$, **** $p < 0.0001$; **D**: Blood lactate before and after exhaustive treadmill running test; **E-I**: Total running distance, duration, maximal speed, heat production, and maximum RER during the $VO_2$max treadmill running test; and **J-K**: Percentage of carbohydrate and fatty acid oxidation, n = 7-9 per group. Data presented as means ± SEM and analyzed by two-way ANOVA. ** $p < 0.01$, **** $p < 0.0001$.

bioRxiv preprint doi: https://doi.org/10.64898/2026.06.18.733224; this version posted June 23, 2026. The copyright holder for this preprint (which was not certified by peer review) is the author/funder, who has granted bioRxiv a license to display the preprint in perpetuity. It is made available under a CC-BY 4.0 International license.

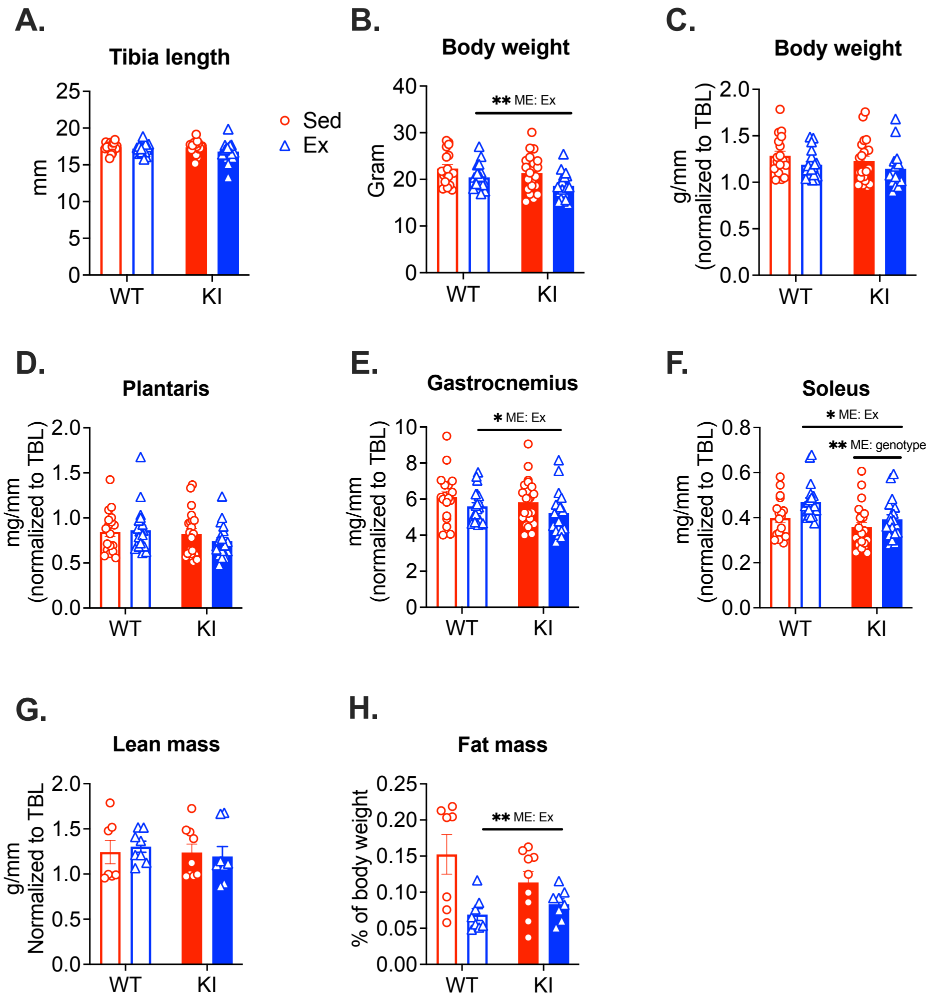


**Supplementary figure 3. Measurements of body weight, muscle mass, lean body mass and fat mass in exercise trained and sedentary WT and *Ampkα2*(T172) KI mice.** *Ampkα2*(T172A) KI and WT littermates were subjected to VWR for 4 weeks with sedentary KI and WT mice as controls followed by direct measurements of tibia length, body weight and muscle mass and echoMRI measurments of lean body mass and fat mass. **A**: Tibia length (TBL); **B-C**: Body weight and body weight normalized by TBL; **D-F**: Skeletal muscle mass nomalized by TBL, n = 18-20 per group; **G-H**: Lean body mass and fat mass, n = 7-9 per group. Data presented as means ± SEM and analyzed by two-way ANOVA. * $p < 0.05$, ** $p < 0.01$.

bioRxiv preprint doi: https://doi.org/10.64898/2026.06.18.733224; this version posted June 23, 2026. The copyright holder for this preprint (which was not certified by peer review) is the author/funder, who has granted bioRxiv a license to display the preprint in perpetuity. It is made available under a CC-BY 4.0 International license.

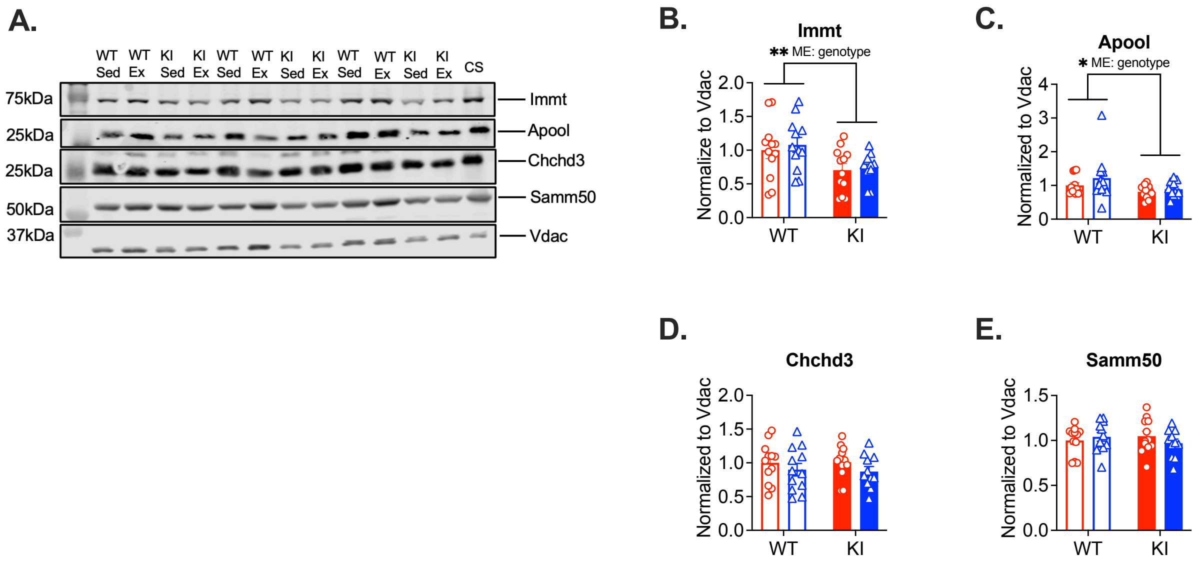


**Supplementary figure 4. Mitochondrial contact site and cristae organizing system (MICOS) proteins are reduced in *Ampkα2*(T172A) mice.** *Ampkα2*(T172A) KI and WT littermates were subjected to VWR for 4 weeks with sedentary KI and WT mice as controls followed by western blot analysis of plantaris muscle. **A**: Representative western blot image for Immt (Mic60), Apool (Mic27), Chchd3 (Mic19) and Samm50; **B-E**: Quantification of protein expression normalized by Vdac, n = 11-12 per group. Data presented as means ± SEM and analyzed by two-way ANOVA are * $p < 0.05$, ** $p < 0.01$.

bioRxiv preprint doi: https://doi.org/10.64898/2026.06.18.733224; this version posted June 23, 2026. The copyright holder for this preprint (which was not certified by peer review) is the author/funder, who has granted bioRxiv a license to display the preprint in perpetuity. It is made available under a CC-BY 4.0 International license.

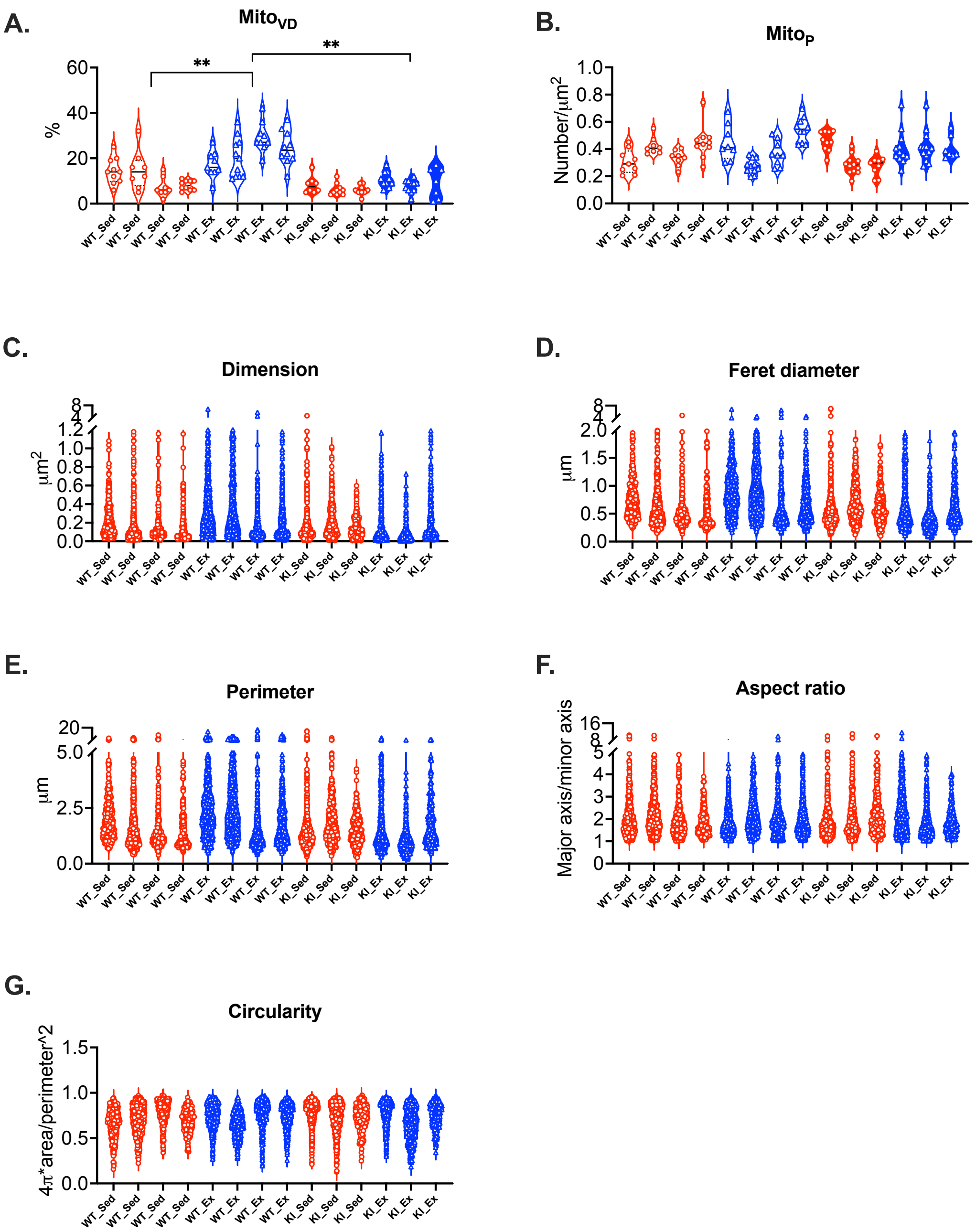


**Supplementary figure 5. Measurements of mitochondrial morphology and structure by TEM in plantaris muscle.** *Ampkα2*(T172A) KI and WT littermates were subjected to VWR for 4 weeks with sedentary KI and WT mice as controls followed by TEM analysis in plantaris muscles. **A-B:** Quantification of mitochondrial volume density ($Mito_{VD}$) and number ($Mito_P$). Each dot denotes a single image analyzed, and each column denotes a single mouse; **C-G**: Mitochondrial morphological measurements (dimension, feret diameter, perimeter, aspect ratio and circularity) from each mouse. Each dot denotes one mitochondrion (350-550 mitochondria quantified per mouse). Data presented as means ± SEM and analyzed by two-way ANOVA. ** $p < 0.01$.

bioRxiv preprint doi: https://doi.org/10.64898/2026.06.18.733224; this version posted June 23, 2026. The copyright holder for this preprint (which was not certified by peer review) is the author/funder, who has granted bioRxiv a license to display the preprint in perpetuity. It is made available under a CC-BY 4.0 International license.

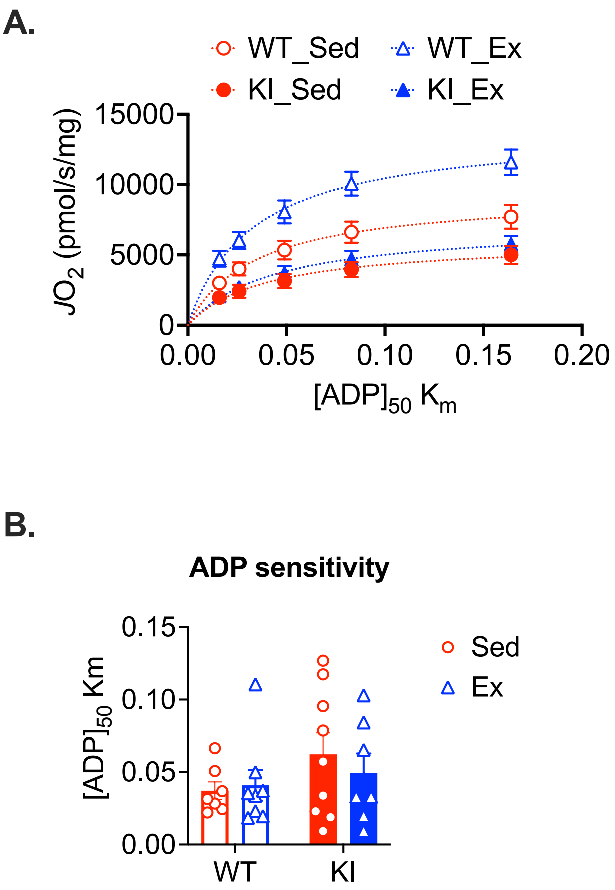


**Supplementary figure 6. ADP sensitivity is not significantly changed by exercise or Ampkα2 (T172).** *Ampkα2*(T172A) KI and WT littermates were subjected to VWR for 4 weeks with sedentary KI and WT mice as controls followed by high-resolution respirometry analysis of isolated mitochondria from plantaris muscles. **A**: Oxygen consumption at ADP [c] 0.016mM, 0.026mM, 0.049mM, 0.083mM, and 0.164mM corresponding to $\Delta G_{ATP}$ of -14.45, -14.12, -13.71, -13.38, and -12.94kCal/mol, respectively; and **B**: ADP sensitivity (Km) determined by Michaelis Menton analysis, n = 7-9 per group. Data presented as means ± SEM.
